# Dynamic behavior and constitutive model of rice husk ash-stabilized loess under freeze–thaw and traffic loading

**DOI:** 10.1371/journal.pone.0361284

**Published:** 2026-10-30

**Authors:** Shaohe Li, Jianfang Wu, Qunxian Lin, Haotian He, Yulong Ma, Bin Wu, Chen Feng

**Affiliations:** 1 Zhejiang Industry Polytechnic College, Shaoxing, Zhejiang, China; 2 Shaoxing University, Shaoxing, Zhejiang, China; 3 Huahui Engineering Design Group Co., Ltd., Shaoxing, Zhejiang, China; 4 Zhejiang Jingwei Investigation Engineering Co., Ltd., Yiwu, Zhejiang, China; Instituto Federal do Espírito Santo: Instituto Federal de Educacao Ciencia e Tecnologia do Espirito Santo, BRAZIL

## Abstract

A series of dynamic triaxial tests was conducted using a global digital system temperature-controlled bidirectional dynamic triaxial system to meet the need for reliable dynamic design parameters of rice husk ash (RHA)-modified loess subgrades in cold regions subjected to the coupled effects of repeated freeze–thaw cycles and traffic-induced dynamic loading. The effects of RHA content and freeze–thaw cycles on dynamic stress–strain behavior, dynamic elastic modulus, normalized dynamic elastic modulus, and damping ratio were systematically investigated. On the basis of the experimental results, a dynamic constitutive model for RHA-modified loess was established. Results indicated that increasing the RHA content substantially enhanced the dynamic stress, dynamic elastic modulus, and normalized dynamic elastic modulus of the modified loess and reduced the damping ratio. Under conditions with coupled freeze–thaw cycling and dynamic loading, the degradation induced by freeze–thaw cycling and traffic loading acted synergistically, resulting in a pronounced deterioration of the dynamic properties of the soil. The findings not only elucidate the improvement mechanism of RHA in the dynamic behavior of loess and the degradation mechanism associated with freeze–thaw cycling but also demonstrate that the proposed constitutive model accurately captures the dynamic response of modified loess under complex loading conditions. This study provides a theoretical basis and technical reference for frost-resistant design, dynamic response analysis, and engineering optimization of loess subgrades in cold regions.

## 1 Introduction

Loess, characterized by a unique genesis and loose structure, is widely distributed in northern and northwestern China. In transportation infrastructure projects, such as railways and highways, loess is extensively used as subgrade fill material [1,2]. However, in cold regions, loess subgrades are subjected to repeated freeze–thaw cycles, which considerably deteriorate their engineering properties and may lead to uneven settlement, strength reduction, and stability loss, thereby threatening the long-term serviceability of transportation infrastructure [3–5]. Meanwhile, the long-term cyclic action of traffic loading further aggravates soil structural damage, accelerating the accumulation of subgrade deterioration [6,7]. Therefore, developing effective improvement methods to enhance the mechanical performance of loess under coupled freeze–thaw cycling and dynamic loading has become a key focus in geotechnical engineering research in cold regions [8].

Conventional improvement methods commonly involve mixing stabilizing agents, such as cement or lime, into soil to enhance its strength and deformation resistance [9,10]. Although this approach is mature and reliable, its high carbon emissions and energy consumption are inconsistent with the current dual-carbon strategy and the concept of green infrastructure development [11]. Therefore, the development of low-carbon and environmentally friendly stabilizing materials has become an urgent engineering challenge. Rice husk ash (RHA) is a by-product of the combustion of rice husk; its global annual production exceeds 120 million tons, and its main component is SiO_2_. It exhibits substantial pozzolanic activity and has a lightweight porous structure and high adsorption capacity. RHA can react with calcium ions in soil to produce secondary cementation, thereby improving soil strength and structural stability [12,13]. Previous studies have shown that incorporating RHA into soil can effectively improve the compressibility, shear strength, and permeability of soil, providing a solid basis for its application in subgrade improvement [14,15].

In recent years, increasing attention has been paid to soil improvement via novel materials. Li et al.[16] conducted dynamic tests on reinforced loess stabilized with RHA and showed that soil exhibits minimal cumulative deformation and optimal strength when the RHA content ranges from 5% to 10%. Qu [17] used cement–gypsum to stabilize peat soil and found that the dynamic strength and deformation resistance of the improved soil are substantially enhanced. Zhuang [18] developed a normalized fitting model of the dynamic shear modulus ratio for loess stabilized with xanthan gum. Liu [19] investigated jute-fiber-reinforced loess and reported that dynamic stress reaches its maximum at a fiber content of 0.3%. Tian [20] stabilized loess by using an enzyme-induced carbonate precipitation solution, demonstrating its effectiveness in enhancing dynamic strength. Gao et al.[21] studied the variability of dynamic elastic modulus in fiber-reinforced loess and found that fiber length, content, and saturation are the primary influencing factors.

With regard to dynamic behavior under freeze–thaw cycles, Sun [22] investigated silt under various sub-zero temperatures and numbers of freeze–thaw cycles and discovered that dynamic stress and dynamic elastic modulus decrease with increasing cycles and stabilize after five cycles. Yan et al.[23] and Yang et al.[24] reported that freeze–thaw cycles considerably affect the dynamic elastic modulus and damping ratio of silt and lightweight stabilized soil, with stabilization trends emerging after six to eight cycles. Tang et al.[25] performed dynamic triaxial tests on loess from Xining and found that the backbone curves stabilize after six freeze–thaw cycles.

With regard to the freeze–thaw dynamic behavior of improved soils, Xu [26] reported that temperature exerts a remarkable effect on the dynamic parameters of lime-stabilized saline soil after freeze–thaw cycles. Song [27] found that the failure vibration number of lime-stabilized subgrade soil is inversely proportional to the confining pressure. Wei [28] established a quantitative relationship among dynamic strength, cyclic loading, and number of freeze–thaw cycles. Fu [29] investigated the influence of delay time on the dynamic strength, dynamic elastic modulus, and excess pore water pressure of cement-stabilized silt under freeze–thaw conditions.

In terms of damage behavior, Qu [30] conducted an in-depth investigation of the mechanical properties and damage mechanisms of moraine soil. Kong and Xie et al.[31,32] established damage models to study the strength characteristics of improved saline soil. The proposed models exhibit good applicability for fractured fine-grained saline soil under low confining pressure.

RHA, an emerging waste-derived material for subgrade soil improvement, is composed primarily of SiO_2_, exhibits high pozzolanic activity, and has a lightweight porous structure and strong adsorption capacity. These characteristics enable RHA to react with calcium ions in the soil through secondary cementitious reactions, thereby enhancing the strength and structural stability of the treated soil.

Although the aforementioned studies have provided valuable insights into the dynamic behavior of improved soils and freeze–thaw effects, several important knowledge gaps remain. (1) Although some studies have investigated the dynamic properties of RHA-modified soils, research on dynamic evolution and damage modeling under coupled freeze–thaw cycles and traffic loading remains scarce. (2) Existing studies on RHA-modified loess have mainly focused on its static mechanical behavior, whereas the coupled freeze–thaw–dynamic interaction mechanism remains insufficiently understood, and the optimal RHA content has rarely been investigated. (3) The mechanisms by which RHA improves the microstructure of loess and inhibits frost heave under freeze–thaw and dynamic loading conditions remain unclear.

To address these knowledge gaps, this study investigated RHA-modified loess by using a global digital system (GDS) temperature-controlled dynamic triaxial system to conduct coupled freeze–thaw and cyclic loading tests. This work aims to fill the research gaps regarding the unclear dynamic evolution behavior under freeze–thaw–dynamic coupling and the absence of an appropriate coupled damage model. The effects of RHA content and freeze–thaw cycles on the dynamic stress–strain relationship, dynamic elastic modulus, and damping ratio of loess were systematically investigated. Meanwhile, scanning electron microscopy (SEM) and X-ray diffraction (XRD) analyses were performed to reveal the physical water adsorption and chemical cementation mechanisms of RHA. On this basis, a coupled damage model integrating the spatially mobilized plane (SMP) criterion and micromechanical statistical damage theory was developed and validated. The proposed model provides a theoretical basis and engineering reference for frost-resistant design and dynamic response analysis of loess subgrades in cold regions.

## 2 Experimental overview and test program

### 2.1 Test apparatus

The experiments were conducted using a GDS temperature-controlled bidirectional dynamic triaxial apparatus. The system has three components: a cooling bath circulation system, a testing system, and a control system. The apparatus can apply a maximum load of 10 kN, with a displacement accuracy of 75 μm. The temperature control range is −30 °C to 60 °C, with a temperature control accuracy of 0.01 °C. The equipment is shown in Fig 1.

**Fig 1:**
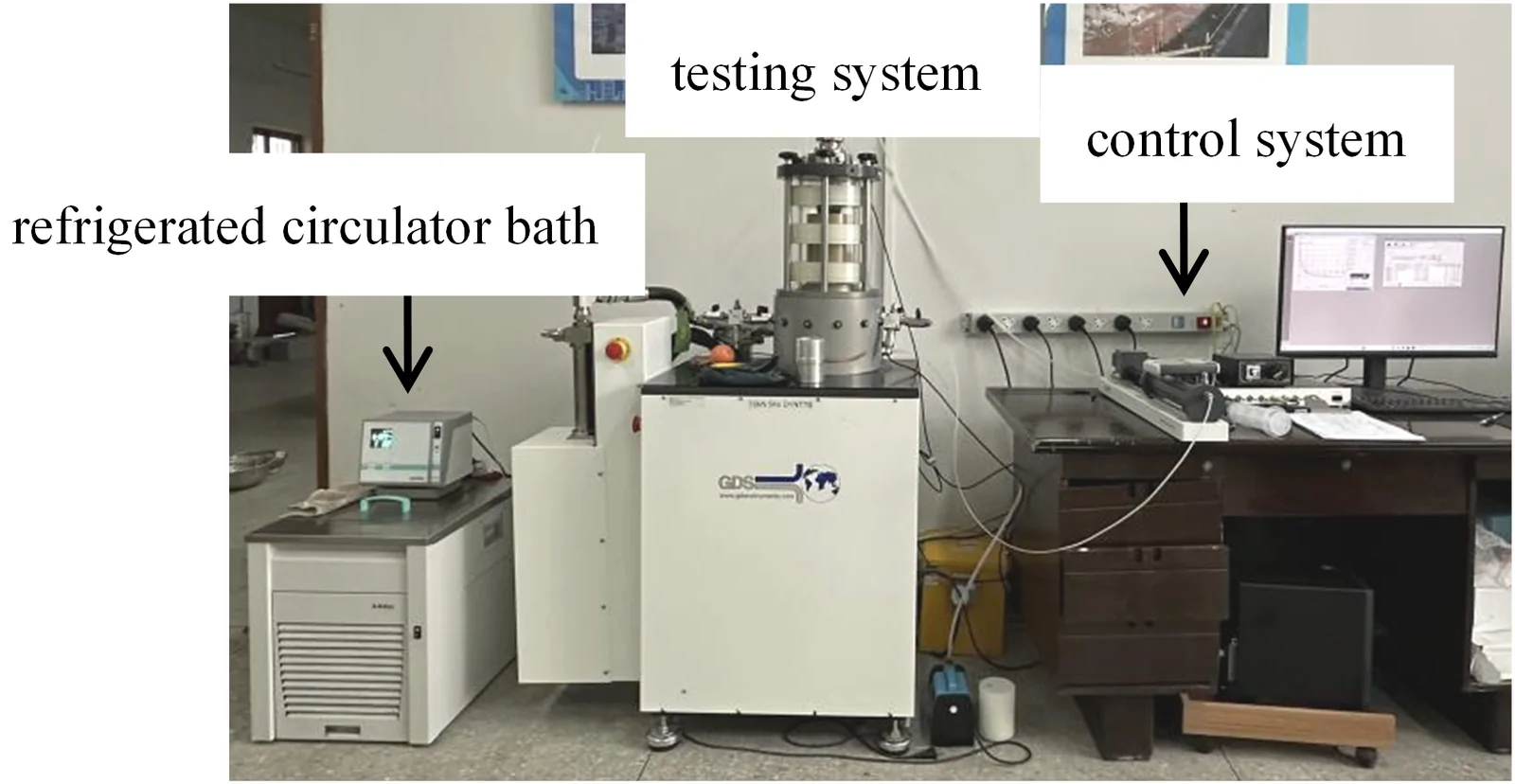
GDS temperature-controlled dynamic triaxial apparatus.

### 2.2 Test materials

The soil samples used in this study were collected from highway subgrade fill in Xifeng City, Gansu Province, and have a light yellow to grayish–yellow color. The soil has a loose structure, with silt-sized particles dominating, and is classified as Q_3_ Malan loess. The basic physical and mechanical properties of the soil are summarized in Table 1. The chemical composition and content of RHA are presented in Table 2. A particle size distribution test was conducted on the soil by using the pycnometer method. The particle size distribution of the soil is shown in Fig 2.

**Table 1:** Basic physical and mechanical properties of the soil.

Soil type	Density (g/cm³)	Water content (%)	Liquid limit (%)	Plastic limit (%)	Plasticity index
Silt	1.7 g/cm^3^	16.1%	25.1	16.5	8.6

**Table 2:** Chemical composition and relative percentages of rice husk ash (%).

Sample	SiO_2_	Al_2_O_3_	CaO	Fe_2_O_3_	MgO	SO_3_
RHA	90	5	2	0.5	0.45	0.2

**Fig 2:**
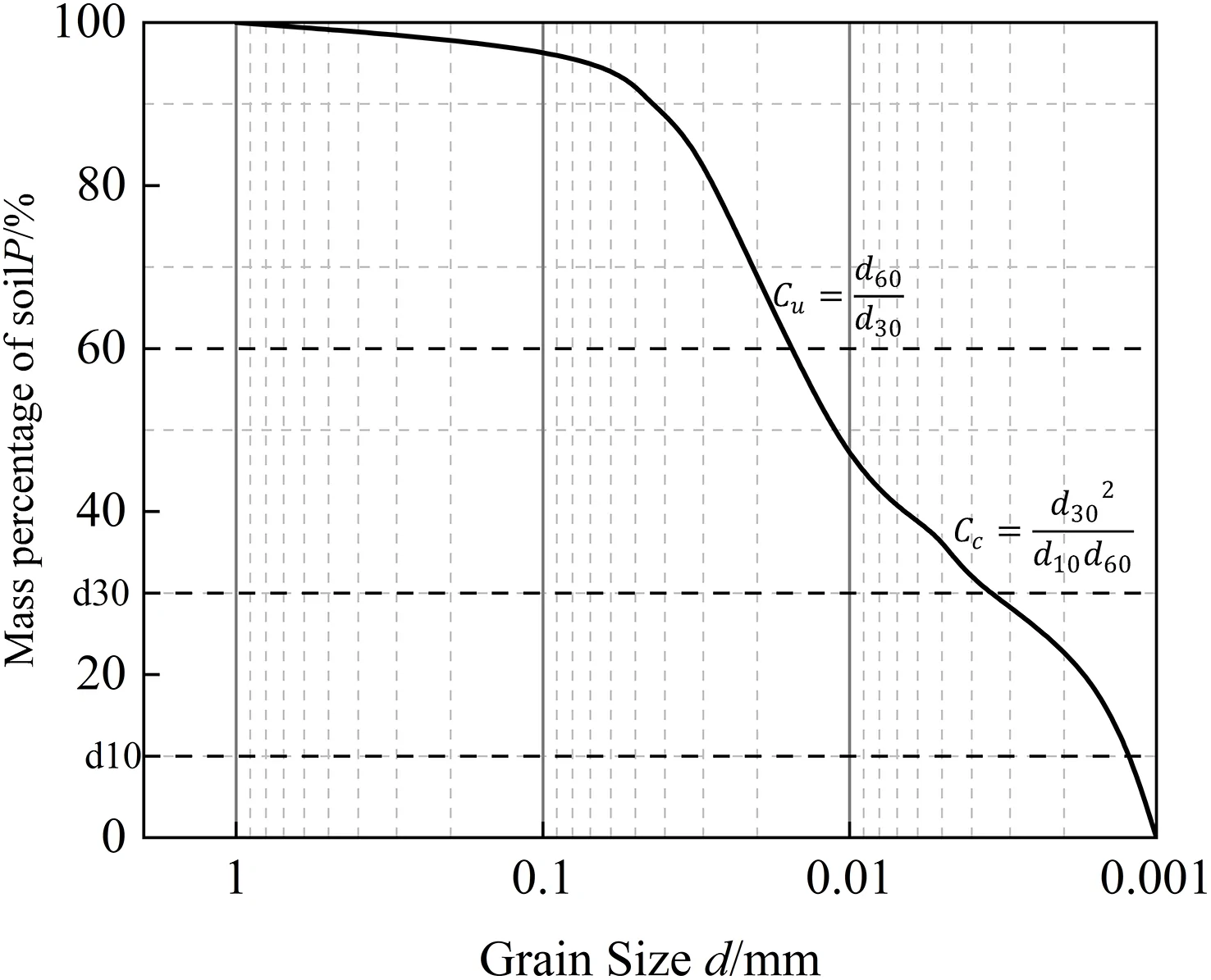
Particle size distribution test on the soil.

Fig 3 presents the results of the compaction test on loess stabilized with varying contents of RHA. As shown in the Fig, the maximum dry density of the loess decreased with increasing RHA content, whereas the optimum moisture content increased. The reduction in maximum dry density became pronounced at high ash contents. In consideration of the influence of dry density on the test results, a uniform dry density of 1.68 g/cm^3^ was adopted throughout the experiments to ensure consistency.

**Fig 3:**
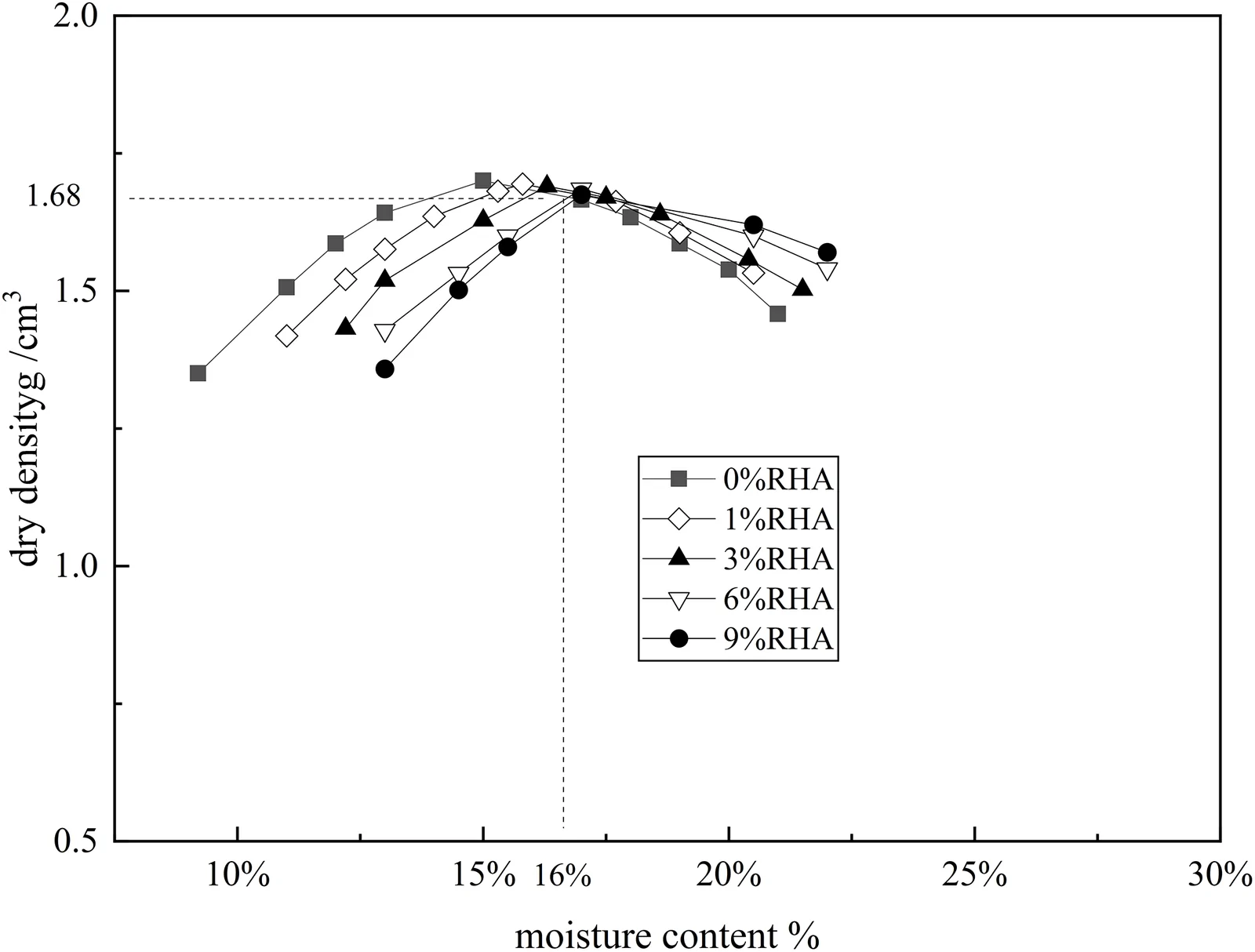
Compaction test on the improved soil.

### 2.3 Test program

The variables in this study included RHA content and number of freeze–thaw cycles. The RHA contents were set to 0%, 1%, 3%, 6%, and 9%. On the basis of the climatic conditions of the Qingyang region, stable minimum and maximum subgrade temperatures, preliminary freeze–thaw test results of three soil samples, and previous studies [22], the freeze–thaw regime was defined with a freezing temperature of −15 °C and a thawing temperature of 25 °C. The number of freeze–thaw cycles (N) was set to 0, 1, 3, 5, and 7. Each specimen was subjected to 12 h of freezing and 12 h of thawing, after which the strain and drainage volume tended to stabilize. Therefore, one freeze–thaw cycle was defined as 12 h of freezing plus 12 h of thawing. These conditions were designed to represent the actual engineering environment of low-traffic loess subgrades on the Qingyang loess plateau. After the freeze–thaw cycles, sinusoidal cyclic loading with a frequency of 1 Hz was applied for 12 loading cycles under an effective confining pressure of 100 kPa. The test was terminated when the axial strain of the specimen reached 5% in accordance with ASTM D3999 (Standard Test Methods for the Determination of Modulus and Damping Properties of Soils Using the Cyclic Triaxial Apparatus). The detailed test program is presented in Table 3.

**Table 3:** Experimental program.

Sample ID	RHA/%	Freeze–thaw temperature /℃	Effective confining pressure /kPa	Loading frequency /Hz	Number of freeze–thaw cycles
1	0	−15/25	100	1	0, 1, 3, 5, 7
2	1				
3	3				
4	6				
5	9				

### 2.4 Sample preparation

The loess samples collected from the field were oven-dried at 105 °C for 8 h in a laboratory. The dried soil was crushed and sieved through a 2 mm mesh. A predetermined mass of dry soil was weighed, and the corresponding amount of RHA was calculated in accordance with the design ratios and thoroughly mixed with the sieved soil. On the basis of the maximum dry density and optimum moisture content obtained from the compaction test, 16% distilled water was added to ensure uniform dry density across all samples. The specimens were sealed and cured at 20 ± 2 °C with a relative humidity of ≥95% for 24 h to ensure sufficient moisture redistribution. Specimens (diameter of 50 mm and height of 100 mm) with a dry density of 1.68 g/cm^3^ were also prepared via layered compaction. After preparation, the specimens were saturated in a vacuum container for more than 48 h. After saturation, the specimens were removed and placed in the testing apparatus for the experiments.

After the tests, representative specimens were selected for SEM analysis. Given the complexity of the coupled freeze–thaw and dynamic triaxial testing procedure, each specimen required 3–7 days to complete. Only one representative specimen was prepared for each testing condition because of the large number of testing variables and experimental constraints. All specimens were prepared in strict accordance with GB/T 50123. The key parameters, including dry density, degree of saturation, and water content, were controlled with an error of less than ±1%. Boundary conditions, such as freeze–thaw temperature, loading frequency, and number of cycles, were kept strictly consistent to minimize systematic experimental errors.

## 3 Results and discussion

### 3.1 Dynamic stress–strain analysis

#### 3.1.1 Effect of rice husk ash content on dynamic stress–strain curves of loess without freeze–thaw cycles.

Fig 4(a) shows the dynamic stress–strain curves of loess stabilized with different RHA contents. The results indicate that under the same dynamic strain, the dynamic stress of loess containing RHA was higher than that of the plain loess without ash. Specifically, dynamic stress increased by approximately 1.7%, 5%, 16%, and 11.6% when 1%, 3%, 6%, and 9% RHA was added, respectively. This result demonstrates that the incorporation of RHA substantially enhanced the dynamic stress-bearing capacity of the soil. Notably, dynamic stress reached its peak at 6% ash content. When the ash content increased to 9%, dynamic stress decreased, with the reduction magnitude placing it between the values for 1% and 3% contents, indicating the existence of an optimal RHA range.

**Fig 4:**
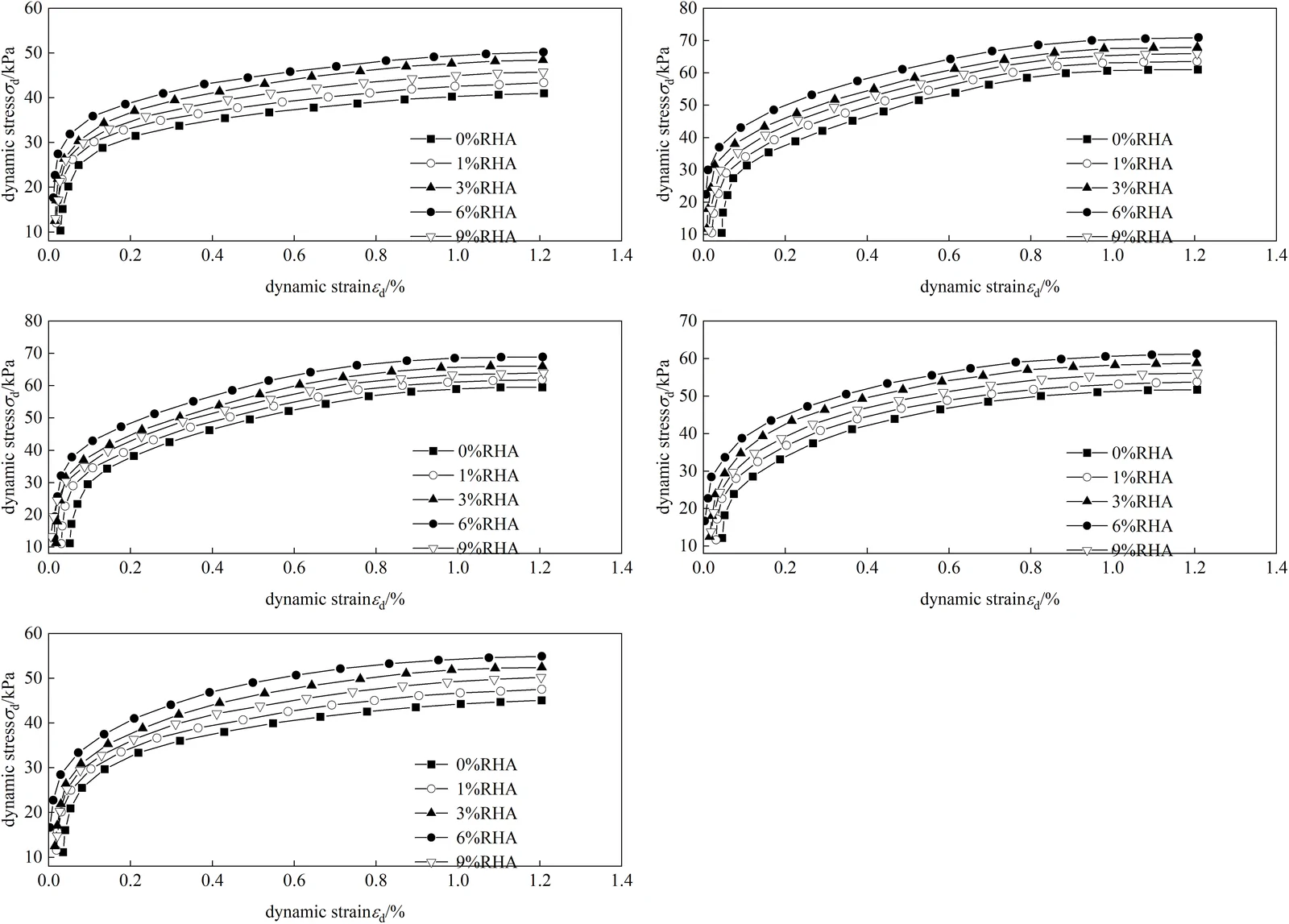
Dynamic stress–strain curve. (a) Variation curve for 0 freeze–thaw cycles, (b) Variation curve for 1 freeze–thaw cycle, (c) Variation curve for 3 freeze–thaw cycles, (d) Variation curve for 5 freeze–thaw cycles, (e)Variation curve for 7 freeze–thaw cycles.

#### 3.1.2 Effect of rice husk ash content on dynamic stress–strain curves of loess after freeze–thaw cycles.

Fig 4(b)–(e) present the dynamic stress–strain relationships of loess with different RHA contents after undergoing freeze–thaw cycles. At the same number of freeze–thaw cycles, the dynamic stress of loess containing RHA was consistently higher than that of plain loess, and the shape of the dynamic stress–strain curves resembled that of the non-freeze–thaw samples. With increasing RHA content, dynamic stress generally increased. However, when the ash content exceeded 6%, dynamic stress reached its maximum. As the ash content further increased, dynamic stress gradually decreased, and at 9% ash content, it fell between the values for 1% and 3% contents, further confirming the existence of an optimal ash threshold.

Furthermore, the curves indicate that at the initial stage of dynamic strain, the loess with different RHA contents exhibited a linear increase in dynamic stress. Within the small strain range, dynamic stress rose rapidly, suggesting that the soil remained at the elastic stage. When dynamic strain exceeded 0.1%, dynamic stress gradually transitioned from linear to nonlinear behavior, and the curves tended to flatten, reflecting the transition of the stabilized soil from elastic to elasto-plastic behavior.

The number of freeze–thaw cycles also affected dynamic stress. In the plain loess without RHA, dynamic stress decreased by approximately 3%, 15%, 10%, and 8% after 1, 3, 5, and 7 freeze–thaw cycles, respectively.

Overall, the trend indicates that a single freeze–thaw cycle has a minor effect on dynamic stress. However, after three cycles, dynamic stress decreases substantially. After five cycles, dynamic stress tends to stabilize, with the reduction rate slowing, indicating that the soil has reached a certain steady state. For each set of tests in this study, 50 consecutive data points were collected for the dynamic stress–strain curves. The curves were smooth without abrupt fluctuations, indicating minimal data scatter. The evolution trends of the measured parameters under different conditions were clearly distinguishable, demonstrating stable and reliable test behavior.

### 3.2 Dynamic elastic modulus analysis

#### 3.2.1 Effect of rice husk ash content on the dynamic elastic modulus of loess without freeze–thaw cycles.

Fig 5(a) shows the relationship between the dynamic elastic modulus and dynamic strain of loess with different RHA contents without freeze–thaw cycles. The results indicate that the dynamic elastic modulus of RHA-modified loess was consistently higher than that of the untreated sample, demonstrating that the incorporation of RHA effectively improved the soil structure and enhanced its dynamic elastic modulus. When the RHA contents were 1%, 3%, 6%, and 9%, the initial dynamic elastic modulus increased by approximately 2.5%, 6.25%, 12.5%, and 10%, respectively. When the RHA contents were 1%, 3%, 6%, and 9%, the initial dynamic elastic modulus increased by about 2.5%, 6.25%, 12.5%, and 10%, respectively.

**Fig 5:**
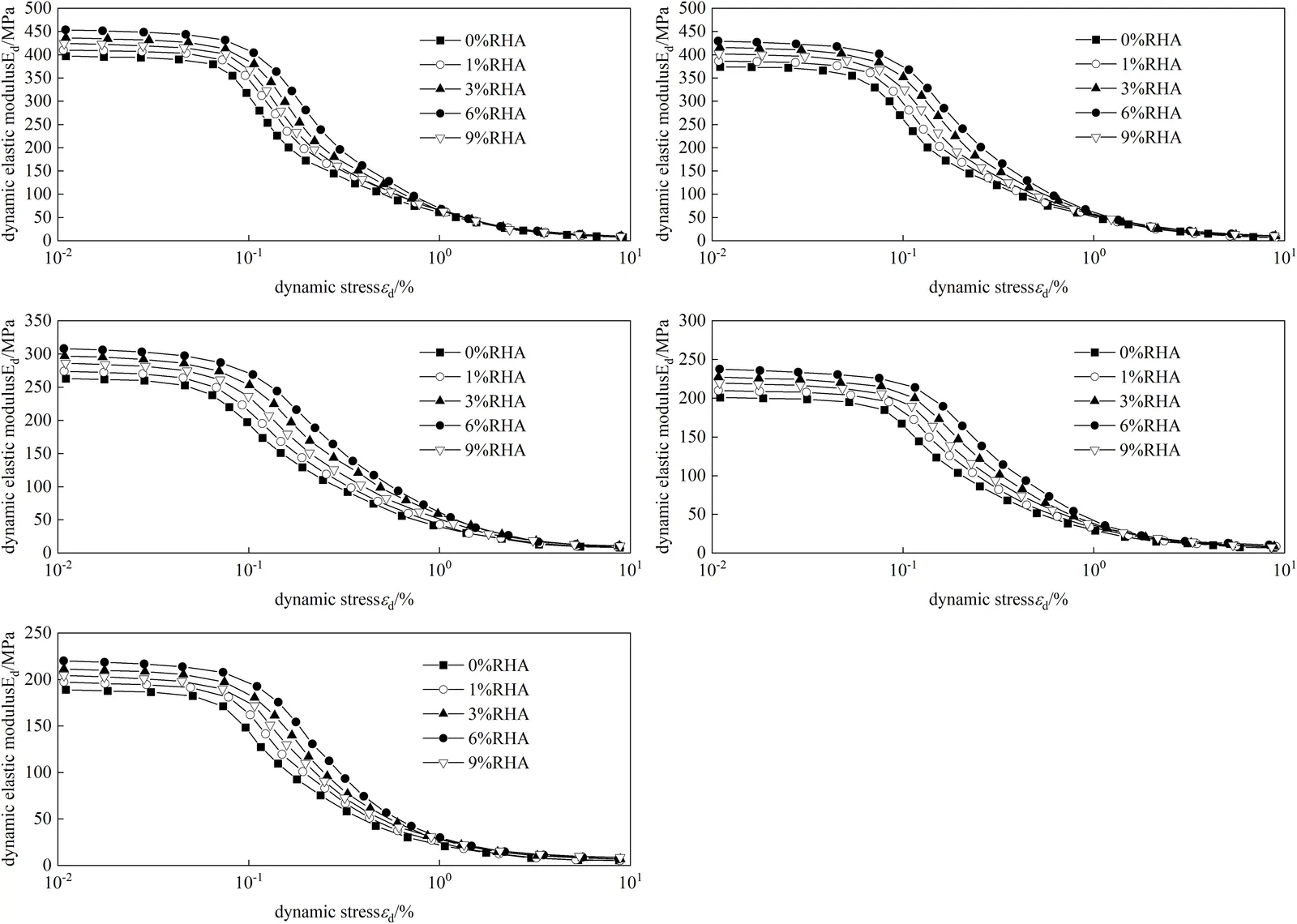
Dynamic elastic modulus–dynamic strain curve. (a) Variation curve for 0 freeze–thaw cycles, (b) Variation curve for 1 freeze–thaw cycle, (c) Variation curve for 3 freeze–thaw cycles, (d) Variation curve for 5 freeze–thaw cycles, (e) Variation curve for 7 freeze–thaw cycles.

Within the dynamic strain range of approximately ${\text{1}\text{0}}^{-\text{2}}$to ${\text{1}\text{0}}^{-\text{1}}$, the dynamic elastic modulus changed slightly, and the curves remained flat. When the dynamic strain exceeded ${\text{1}\text{0}}^{-\text{1}}$, the dynamic elastic modulus decreased considerably with increasing dynamic strain. When the dynamic strain reached approximately ${\text{1}\text{0}}^{\text{0}}$, the dynamic elastic modulus stabilized, and the curves corresponding to different RHA contents gradually converged, indicating that the soil entered a stable nonlinear stage.

#### 3.2.2 Effect of rice husk ash content on the dynamic elastic modulus of loess after freeze–thaw cycles.

Fig 5(b)–(e) show the relationships between the dynamic elastic modulus and dynamic strain of loess with different RHA contents after 1, 3, 5, and 7 freeze–thaw cycles, respectively. At the same number of freeze–thaw cycles, the dynamic elastic modulus of the soil gradually increased with increasing RHA content and reached the maximum at an RHA content of 6%. When the RHA content continued to increase, the dynamic elastic modulus decreased.

At the same dynamic strain, an increase in the number of freeze–thaw cycles led to a large reduction in the dynamic elastic modulus. For the loess without RHA, the initial dynamic elastic modulus decreased by approximately 3.7%, 35%, 50%, and 55% after 1, 3, 5, and 7 freeze–thaw cycles, respectively. The data indicate that one freeze–thaw cycle had a minor effect on the dynamic elastic modulus. However, starting from three cycles, the modulus decreased substantially, and after five cycles, it stabilized. This finding demonstrates that the freeze–thaw cycles caused notable degradation of the dynamic elastic modulus of loess, which became less pronounced after five cycles.

### 3.3 Analysis of dynamic elastic modulus ratio

Fig 6(a)–(e) illustrate the relationships between the dynamic elastic modulus ratio and dynamic strain of loess containing different RHA contents after 0, 1, 3, 5, and 7 freeze–thaw cycles. Fig 6(a) shows that for specimens without freeze–thaw cycles, the dynamic elastic modulus ratio decreased considerably with increasing RHA content. At a dynamic strain of approximately 10 ⁻ ^1^, the dynamic elastic modulus ratio of the untreated loess was about 0.71, whereas that of the specimen containing 6% RHA was only 0.30, which is less than half that of the untreated sample. This result demonstrates that RHA incorporation effectively reduced the dynamic elastic modulus ratio of loess.

**Fig 6:**
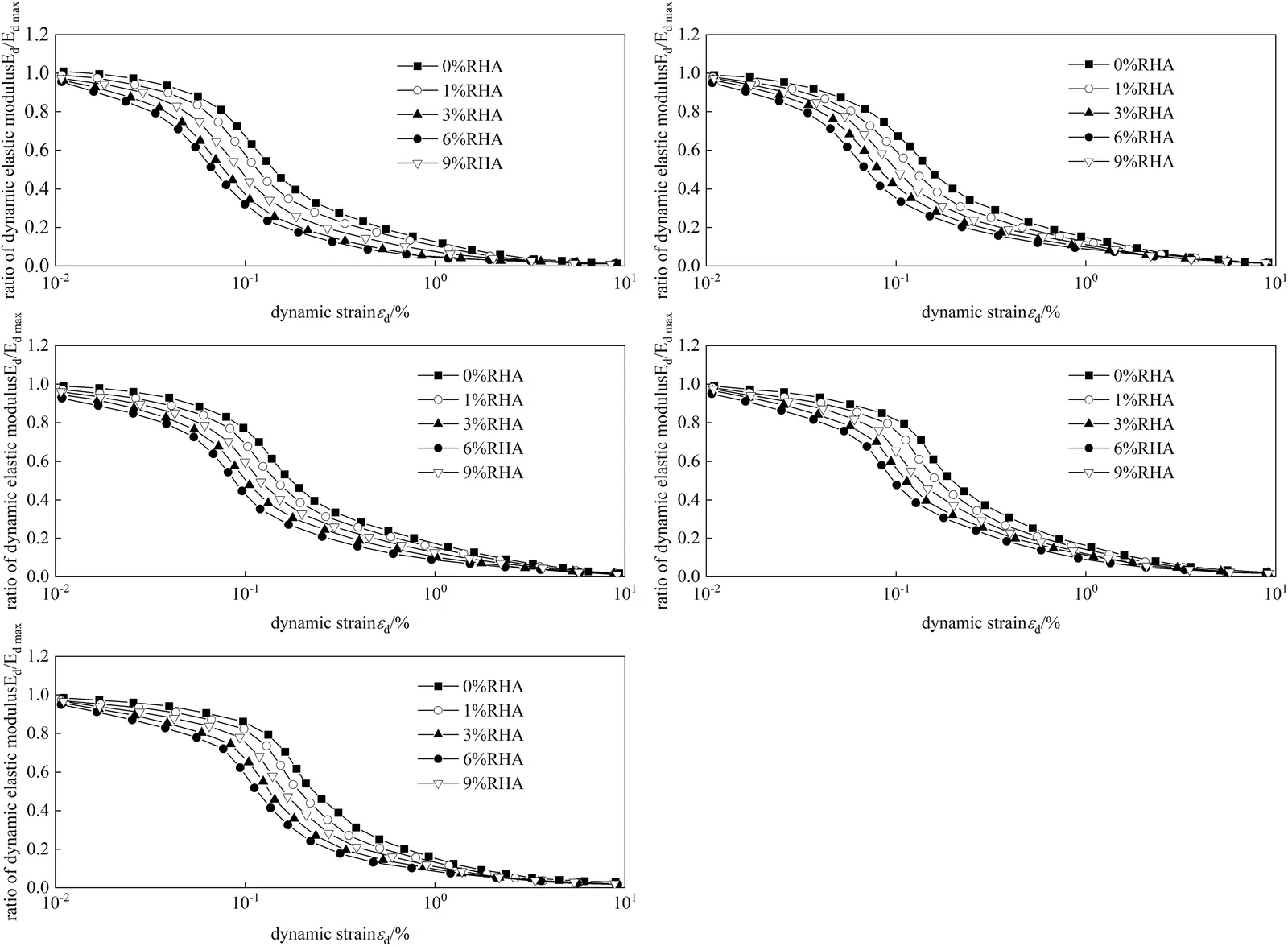
Dynamic elastic modulus ratio–dynamic strain curve. (a) Variation curve for 0 freeze–thaw cycles, (b) Variation curve for 1 freeze–thaw cycle, (c) Variation curve for 3 freeze–thaw cycles, (d) Variation curve for 5 freeze–thaw cycles, (e) Variation curve for 7 freeze–thaw cycles.

Furthermore, the dynamic elastic modulus ratio decreased continuously with increasing RHA content and reached the minimum value at 6%. When the content increased from 6% to 9%, the dynamic elastic modulus ratio slightly increased. This trend indicates that the optimal RHA content in loess is approximately 6%, beyond which the improvement gradually weakens.

As shown in Fig 6(b)–(e), at the same number of freeze–thaw cycles, the variation trend of the dynamic elastic modulus ratio of loess with different RHA contents was generally consistent with that observed in the specimens without freeze–thaw cycles, indicating the presence of an optimal ash content. The Fig also shows that the dynamic elastic modulus ratio changed only slightly within the small strain range (approximately 10 ⁻ ^2^). As the dynamic strain increased, the dynamic elastic modulus ratio began to decrease substantially. When the dynamic elastic modulus reached a large value, the decrease in the dynamic elastic modulus ratio gradually stabilized, and the curves eventually converged, indicating that the soil had entered a stable nonlinear stage.

### 3.4 Damping ratio analysis

Fig 7(a)–(e) show the relationships between damping ratio and dynamic strain for loess with different rice husk ash contents after 0, 1, 3, 5, and 7 freeze–thaw cycles.At low dynamic stress amplitudes, the damping ratio increases slowly. As dynamic strain increases, it rises rapidly before gradually approaching a plateau at higher strain levels.

**Fig 7:**
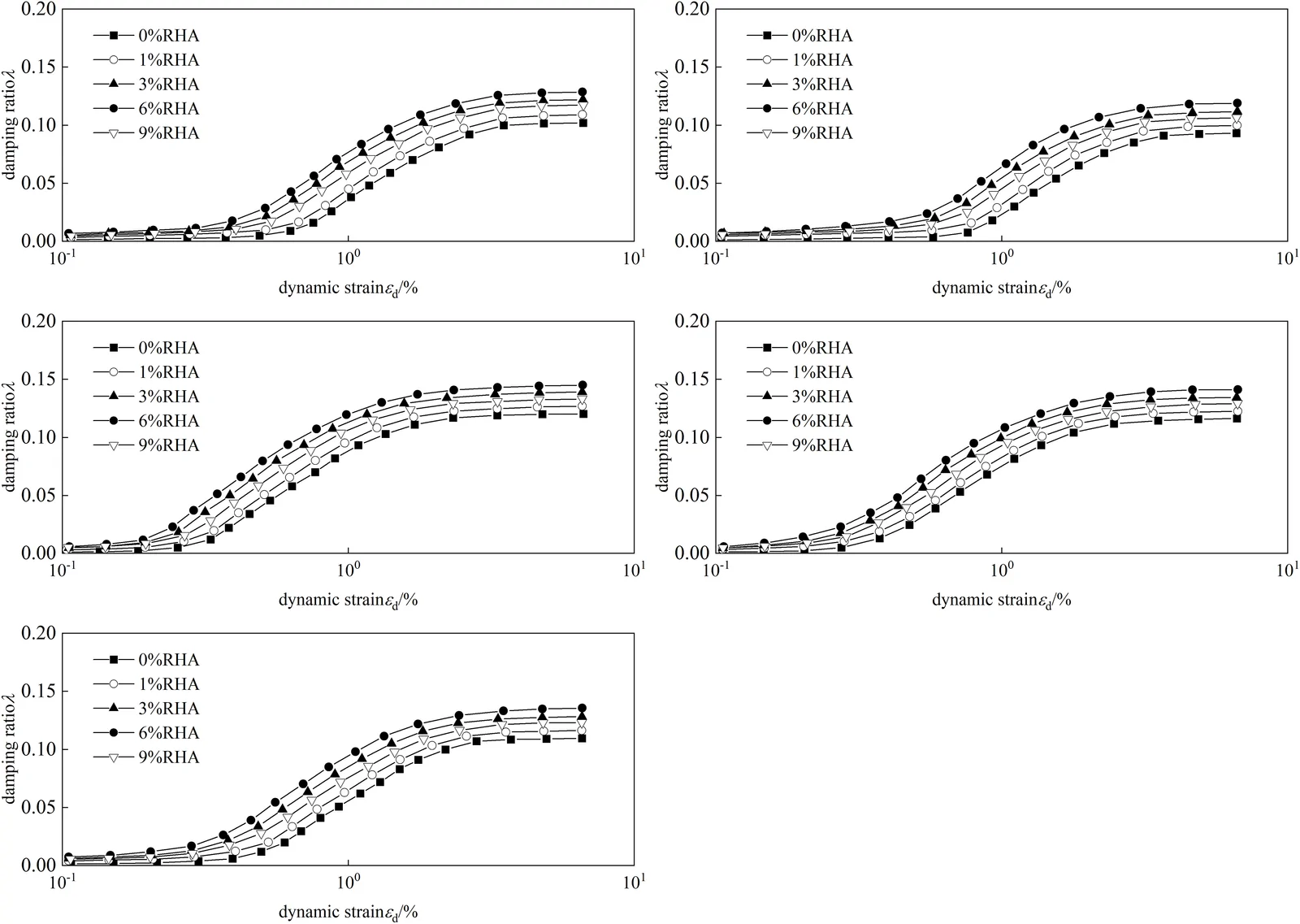
Damping ratio–dynamic strain curve. (a) ariation curve for 0 freeze–thaw cycles (b) ariation curve for 1 freeze–thaw cycles, (c) ariation curve for 3 freeze–thaw cycles (d) ariation curve for 5 freeze–thaw cycles, (e) ariation curve for 7 freeze–thaw cycles.

At a given number of freeze–thaw cycles, the damping ratio initially increased with increasing rice husk ash content, reached a maximum at approximately 6%, and then decreased at higher contents.At a given dynamic strain, the damping ratio decreased as the number of freeze–thaw cycles increased.This decrease may be attributed to progressive loosening of the soil structure and weakened interparticle interactions caused by repeated frost heave and thaw settlement, which may reduce the soil’s capacity to dissipate energy.

## 4 Determination of the maximum dynamic elastic modulus correction coefficient

In accordance with the definition of the dynamic elastic modulus, the following relationship can be obtained.


$$\begin{array}{c} E_{d}=\frac{\sigma_{d}}{\varepsilon_{d}}=\frac{\text{1}}{a+b\varepsilon_{d}} \end{array}$$ (1)

The initial dynamic elastic modulus, $E_{d\text{0}}$, of loess without RHA at zero freeze–thaw cycles can be obtained from the stress–strain relationship curve. After 1, 3, 5, and 7 freeze–thaw cycles, the dynamic elastic modulus exhibited different degrees of degradation. In this study, the maximum dynamic elastic modulus of the soil after different numbers of freeze–thaw cycles was analyzed and fitted via regression analysis. The fitted curves are shown in Fig 8, and the fitting model can be expressed as follows:

**Fig 8:**
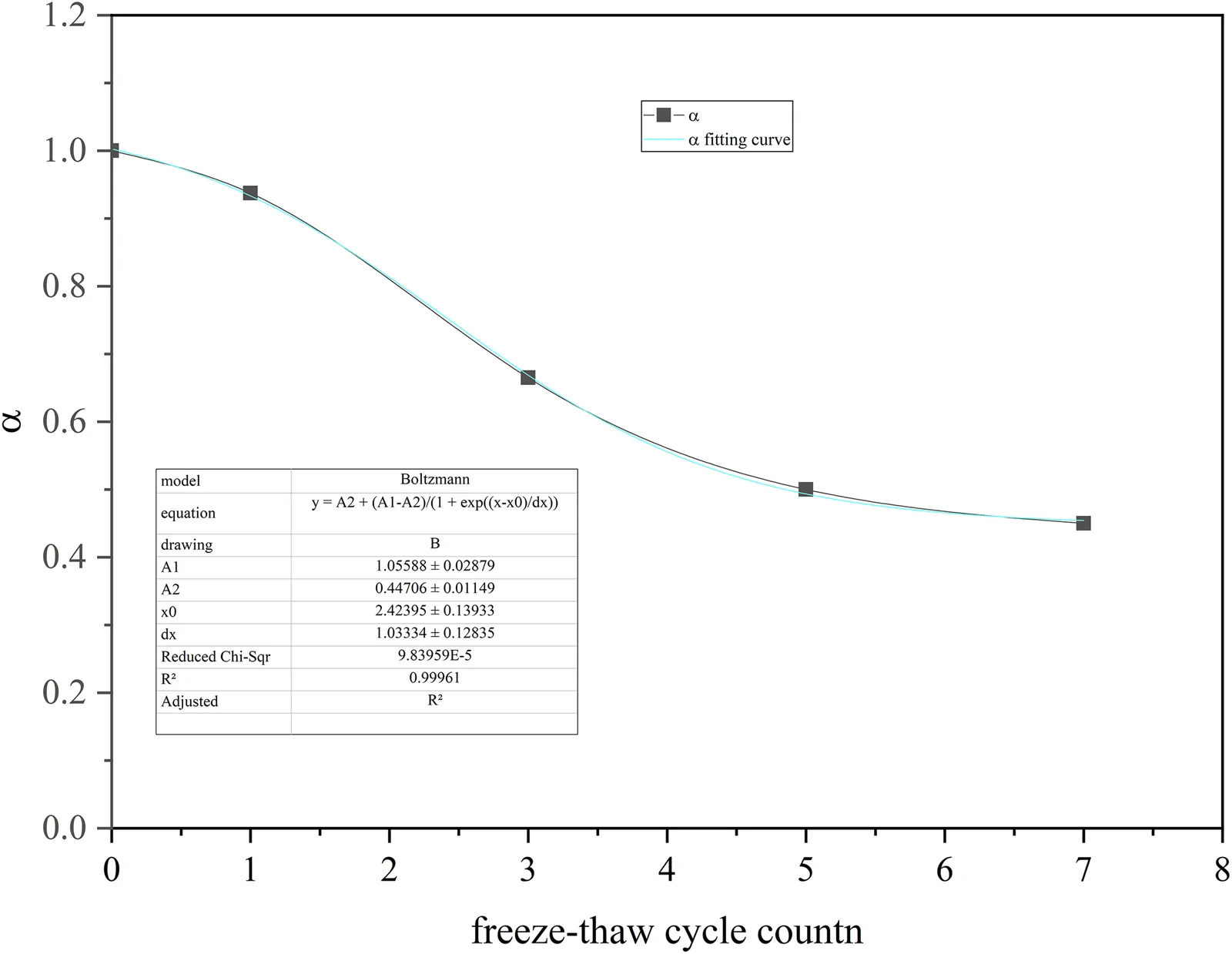
Variation in the maximum dynamic elastic modulus.


$$\begin{array}{c} \alpha (N)=\text{0}.\text{4}\text{4}\text{7}\text{0}\text{6}+\text{0}.\text{6}\text{0}\text{8}\text{8}\text{2}/(\text{1}+\text{exp}(\frac{N-\text{2}.\text{4}\text{2}\text{3}\text{9}}{\text{1}.\text{0}\text{3}\text{3}})\text{,} \end{array}$$ (2)

where α(N)is the correction factor for the maximum dynamic elastic modulus of loess without RHA under Nfreeze–thaw cycles and Ndenotes the number of freeze–thaw cycles.

The initial dynamic elastic modulus of the soil changed when RHA was incorporated even at zero freeze–thaw cycles. The initial dynamic elastic modulus of soils with different RHA contents was analyzed and fitted using regression analysis. The fitted curves are presented in Fig 9. The fitting model can be expressed as follows:

**Fig 9:**
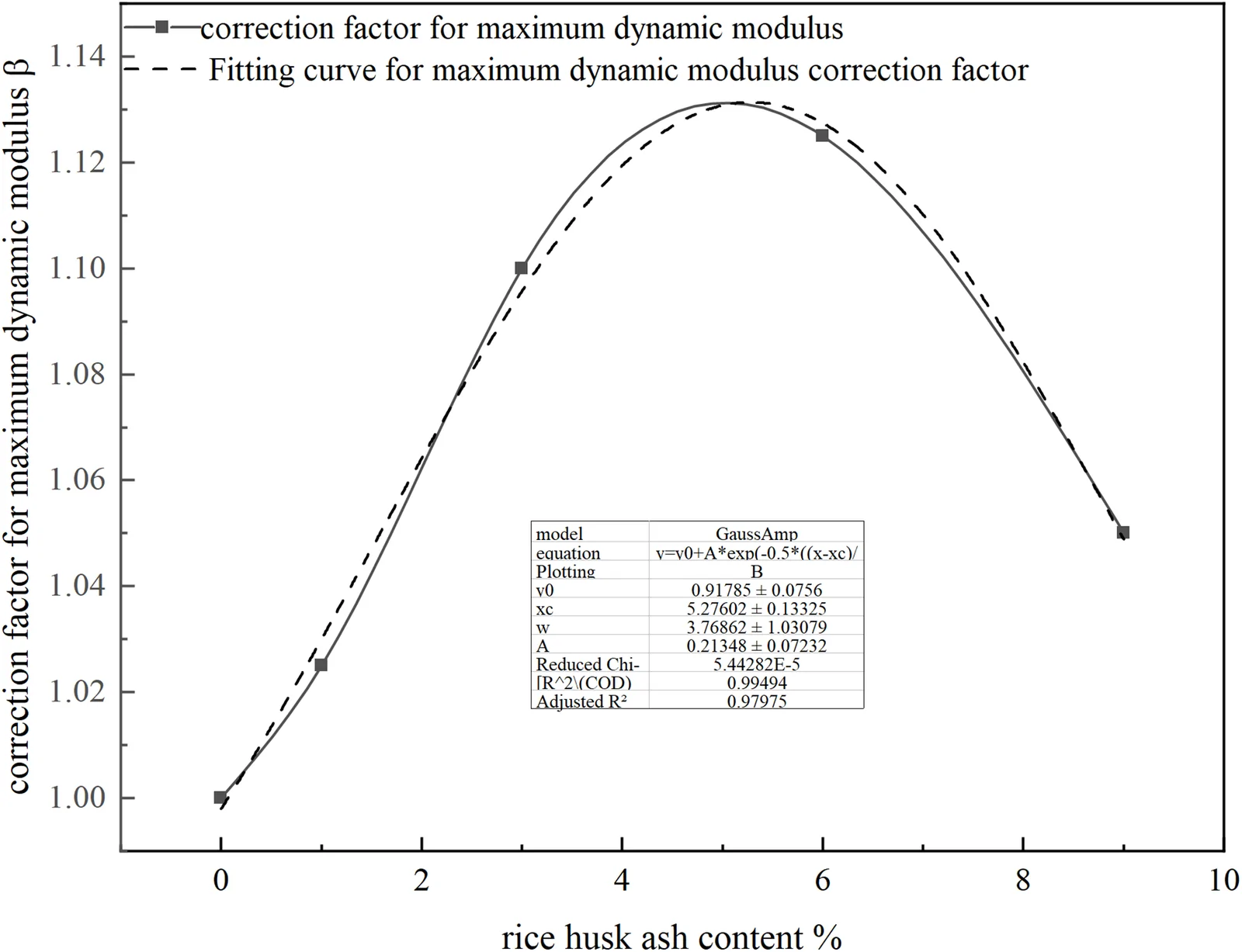
Variation in the maximum dynamic elastic correction with freeze–thaw cycles modulus correction with RHA content.


$$\begin{array}{c} \beta (A)=\text{0}.\text{9}\text{1}\text{7}+\text{0}.\text{2}\text{1}\text{3}\cdot \text{exp}{\left(-\text{0}.\text{5}\cdot (\frac{A-\text{5}.\text{2}\text{7}}{\text{3}.\text{7}\text{6}\text{8}}\right)}^{\text{2}}), \end{array}$$ (3)

where $\beta_{A}$is the correction coefficient of the initial dynamic elastic modulus related to RHA content and Adenotes RHA content.

## 5 Establishment of the damage model

### 5.1 Model development

1. **Combined coefficient for rice husk ash improvement and freeze–thaw damage**

On the basis of the relative increase in peak dynamic stress, a combined correction coefficient, $k_{dw}$, was defined to consider the effects of the number of freeze–thaw cycles (N) and RHA content (A). The expression can be written as follows:


$$\begin{array}{c} k_{dw}=\alpha (N)\beta (A)\text{.} \end{array}$$ (4)

Accordingly, the dynamic elastic modulus of loess without RHA under freeze–thaw cycles, $E_{dn}$, can be expressed as


$$\begin{array}{c} {\text{E}}_{dn}={\text{E}}_{d\text{0}}\alpha (\text{N})\text{,} \end{array}$$ (5)

where $E_{d\text{0}}$ denotes the initial dynamic elastic modulus of the soil before any freeze–thaw cycle. The maximum dynamic elastic modulus of loess containing a certain amount of RHA, $E_{dA,N}$, is expressed as


$$\begin{array}{c} {\text{E}}_{d}(\text{A},\text{N})={\text{E}}_{d\text{0}}\alpha (\text{N})\beta (\text{A})\text{.} \end{array}$$ (6)

According to Lemaitre’s equivalent strain hypothesis [33], the strain induced by effective stress in an undamaged material is equivalent to the strain induced by nominal stress in a damaged material. Damage variable $D_{n}$is defined as follows:


$$\begin{array}{c} D_{n}=\text{1}-\frac{E_{dn}}{E_{d\text{0}}}\text{.} \end{array}$$ (7)

Substituting Eq. (5) into Eq. (7) yields


$$\begin{array}{c} {\text{D}}_{\text{n}}=\text{1}-\alpha (\text{N})\text{.} \end{array}$$ (8)

The improved coupled damage model is expressed as


$$\begin{array}{c} D_{total}=D_{n}+D-{k_{dw}D}_{n}D\text{.} \end{array}$$ (9)

On the basis of Lemaitre’s equivalent strain hypothesis, Eq. (9) can be rewritten as


$$\begin{array}{c} {\text{D}}_{\text{total}}=\text{1}-\alpha (\text{N})\beta (\text{A})\text{exp}\left[-{\left(\frac{{\text{F}}_{d}}{{\text{K}}_{d}}\right)}^{m}\right]\text{.} \end{array}$$ (10)

By using the dynamic SMP spatial sliding surface criterion and considering the pseudo-triaxial dynamic loading condition ($\sigma_{\text{2}}=\sigma_{\text{3}}=\text{100}\;\text{kPa}$), the dynamic strength variable of the microelement is defined as


$$\begin{array}{c} F_{d}=\frac{\sigma_{d}^{*}+\sigma_{\text{0}}^{d}}{\sigma_{\text{3}}+\sigma_{\text{0}}^{d}}\text{,} \end{array}$$ (11)

where $F_{d}$is the dynamic strength variable of the microelement, $\sigma_{d}^{*}$is the effective dynamic deviatoric stress, and $\sigma_{\text{0}}^{d}=c_{d}\text{cos}\varphi_{d}$represents the dynamic cohesive stress.

In accordance with generalized Hooke’s law, the relationship between dynamic deviatoric stress and strain can be written as


$$\begin{array}{c} \varepsilon_{d}=\frac{\sigma_{d}^{*}}{E_{dn}}\text{.} \end{array}$$ (12)

Substituting Eq. (5) into Eq. (12) yields


$$\begin{array}{c} \sigma_{d}^{*}=E_{d\text{0}}\alpha (N)\varepsilon_{d}\text{.} \end{array}$$ (13)

By substituting Eq. (13) into Eq. (11) and simultaneously considering the strengthening effect of RHA on the soil microstructure, the following relationship can be obtained.


$$\begin{array}{c} F_{d}=\frac{\beta (A)E_{d\text{0}}\alpha (N)\varepsilon_{d}+\sigma_{\text{0}}^{d}}{\sigma_{\text{3}}+\sigma_{\text{0}}^{d}} \end{array}$$ (14)

On the basis of Lemaitre’s damage theory, we have


$$\begin{array}{c} \sigma_{d}=E_{d\text{0}}\varepsilon_{d}\cdot (\text{1}-D_{total})\text{.} \end{array}$$ (15)

Substituting Eq. (10) into Eq. (15) yields


$$\begin{array}{c} \sigma_{d}=E_{d\text{0}}\varepsilon_{d}\alpha (N)\beta (A)exp\left[-({\frac{F_{d}}{K_{d}})}^{m}\right]\text{.} \end{array}$$ (16)

By substituting Eqs. (4) and (14) into Eq. (16) and performing algebraic simplification, the core equation of the dynamic statistical damage constitutive model for RHA-stabilized loess under freeze–thaw cycles is obtained as


$$\begin{array}{c} \sigma_{d}=k_{dw}\cdot E_{dn}\cdot \varepsilon_{\text{1}d}\cdot exp\left[-{\left(\frac{k_{dw}\cdot E_{dn}\cdot \varepsilon_{\text{1}d}+\sigma_{\text{0}}^{d}}{(\sigma_{\text{3}}+\sigma_{\text{0}}^{d})\cdot K_{d}}\right)}^{m}\right]. \end{array}$$ (17)

After transforming the model in Eq. (17) and applying a double logarithmic transformation, we define the linear variable as


$$\begin{array}{c} n\left[-ln\left(\frac{\sigma_{d}}{k_{dw}E_{dn}\varepsilon_{d}}\right)\right]=mln(\frac{k_{dw}E_{dn}\varepsilon_{d}+\sigma_{\text{0}}^{d}}{\sigma_{\text{3}}+\sigma_{\text{0}}^{d}})-m\cdot lnK_{d}\text{,} \end{array}$$ (18)

$$\begin{array}{c} y=ln[-ln(\frac{\sigma_{d}}{k_{dw}E_{dn}\varepsilon_{d}})]\text{,} \end{array}$$ (19)

$$\begin{array}{c} x=ln(\frac{k_{dw}E_{dn}\varepsilon_{d}+\sigma_{\text{0}}^{d}}{\sigma_{\text{3}}+\sigma_{\text{0}}^{d}})\text{,} \end{array}$$ (20)

$$\begin{array}{c} b=-m\cdot lnK_{d}\text{.} \end{array}$$ (21)

The resulting linear equation is


$$\begin{array}{c} y=m\cdot x+b\text{.} \end{array}$$ (22)

Through the least squares method, the linear fit yields parameters mand b. The relationship for bcan be expressed as


$$\begin{array}{c} K_{d}=\text{exp}(-\frac{b}{m})\text{.} \end{array}$$ (23)

### 5.2 Model validation

On the basis of the derivation of the freeze–thaw damage model, a constitutive model for RHA-stabilized loess under freeze–thaw cycles was established. The test results for two extreme freeze–thaw cycles (0 and 7 cycles) were used to derive the model parameters in accordance with the experimental data and the derived equations, and the calculated results are presented in Tables 4 and 5. The model was then validated for RHA contents of plain soil, namely, 1%, 3%, 6%, and 9%, under both 0- and 7-cycle freeze–thaw conditions, and the validation results are given in Fig 10.

**Table 4:** Constitutive model parameters of plain loess under freeze–thaw damage.

Freeze–thaw cycles	Initial dynamic elastic modulus $E_{d0}$(MPa)	Poisson’s ratio ν	Cohesion $\sigma_{c}$(kPa)	$K_{d}$	m
0	397	0.34	68.0	3.45	1.72
1	375	0.33	64.0	3.9	1.65
3	261	0.32	60.5	4.5	1.57
5	203	0.31	55.5	5.20	1.47
7	186	0.30	51.0	5.80	1.40

**Table 5:** Constitutive model parameters of loess stabilized with 6% RHA under freeze–thaw damage.

Freeze–thaw cycles	Initial dynamic elastic modulus $E_{d0}$(MPa)	Poisson’s ratio ν	Cohesion $\sigma_{c}$(kPa)	$K_{d}$	m
0	450	0.36	68.0	3.6	1.68
1	425	0.35	64.0	4.10	1.60
3	310	0.34	60.0	4.70	1.52
5	242	0.33	56.0	5.30	1.44
7	225	0.32	52.0	5.90	1.36

**Fig 10:**
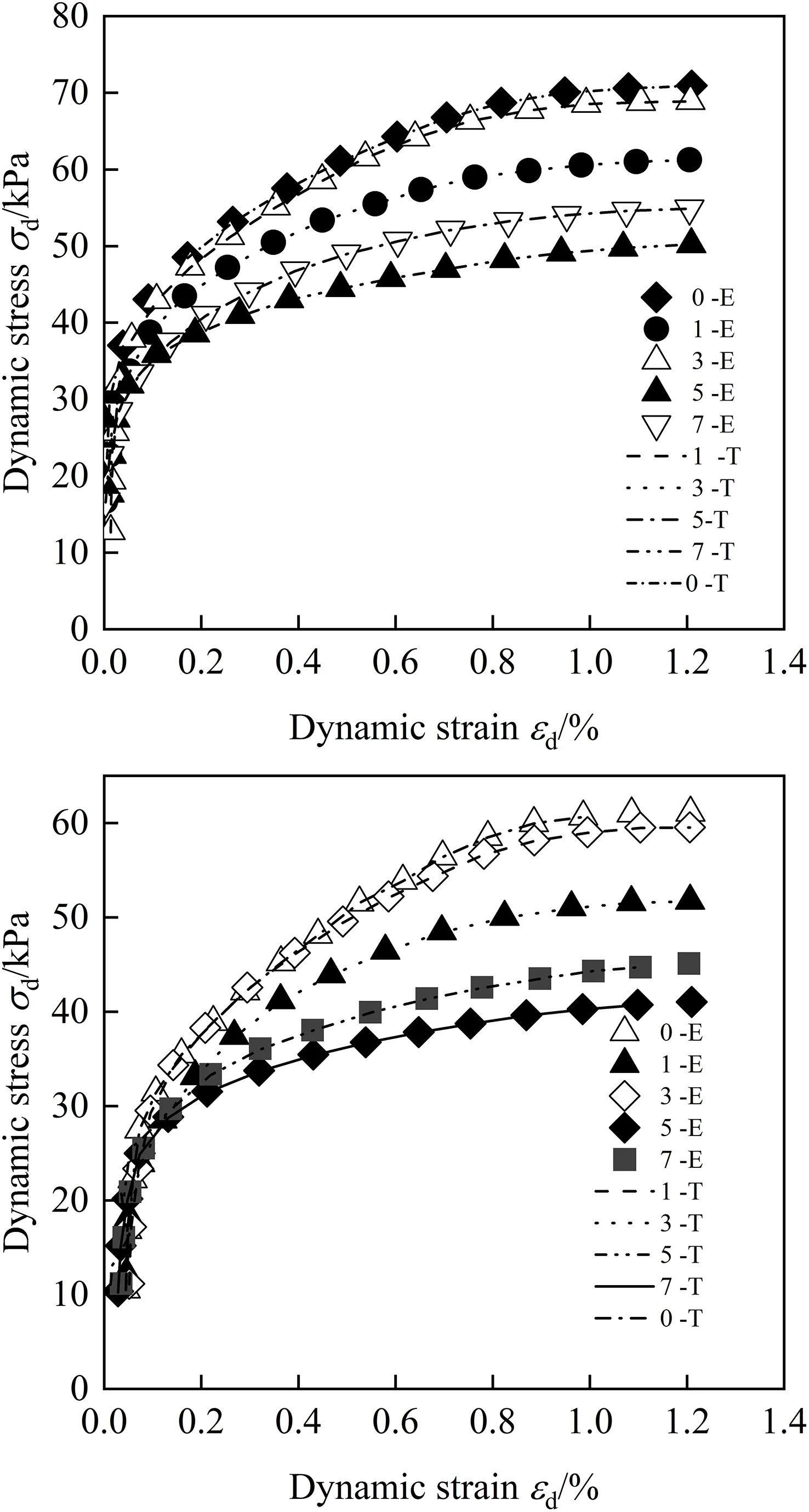
Verification of constitutive model results. (a) Loess with 0% RHA. (b) Loess with 6% RHA.

As shown in Table 4, the incorporation of 6% RHA had a remarkable effect on the damage parameters of loess. $K_{d}$ slightly increased, enhancing the initial resistance to damage, whereas *m* slightly decreased, reducing the nonlinearity of damage evolution. This effect is primarily attributed to the filling–cementation mechanism, which improves the internal structure of the soil and enhances its initial structural stability.

For plain loess (0% RHA) and loess stabilized with 6% RHA, the *m* value gradually decreased with increasing freeze–thaw cycles, indicating enhanced soil brittleness, whereas $K_{d}$ exhibited the opposite trend. The monotonic decrease in *m* reflects a gradual damage evolution, which is consistent with the cumulative damage effects of the freeze–thaw, swelling–settlement process. The increase in $K_{d}$ aligns well with the accumulated freeze–thaw damage. Analysis of the parameters showed that RHA effectively improved the freeze–thaw durability of loess. The $K_{d}$ increment for 6% RHA loess was slightly smaller than that for plain loess, indicating suppressed damage accumulation, and the *m* value remained consistently small, suggesting uniform damage evolution and enhanced structural stability.

The coupled constitutive model derived in this study was used to calculate the theoretical damage values for plain loess (0% RHA) and loess stabilized with 6% RHA under 0, 1, 3, 5, and 7 freeze–thaw cycles, as shown in Figs 11 and 12. The calculated values were consistent with the observed freeze–thaw damage behavior of loess.

**Fig 11:**
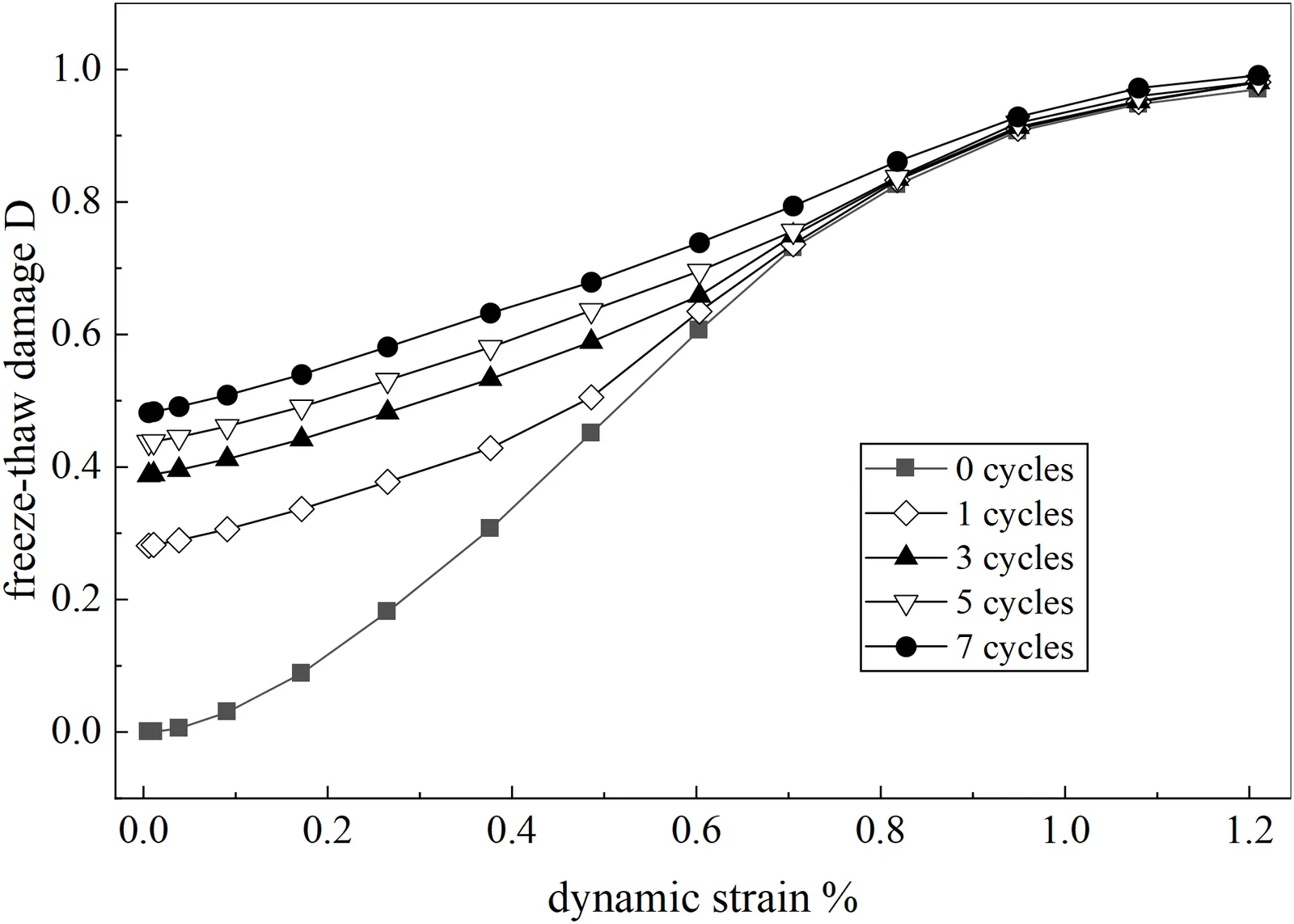
Damage evolution of loess with 0% RHA.

**Fig 12:**
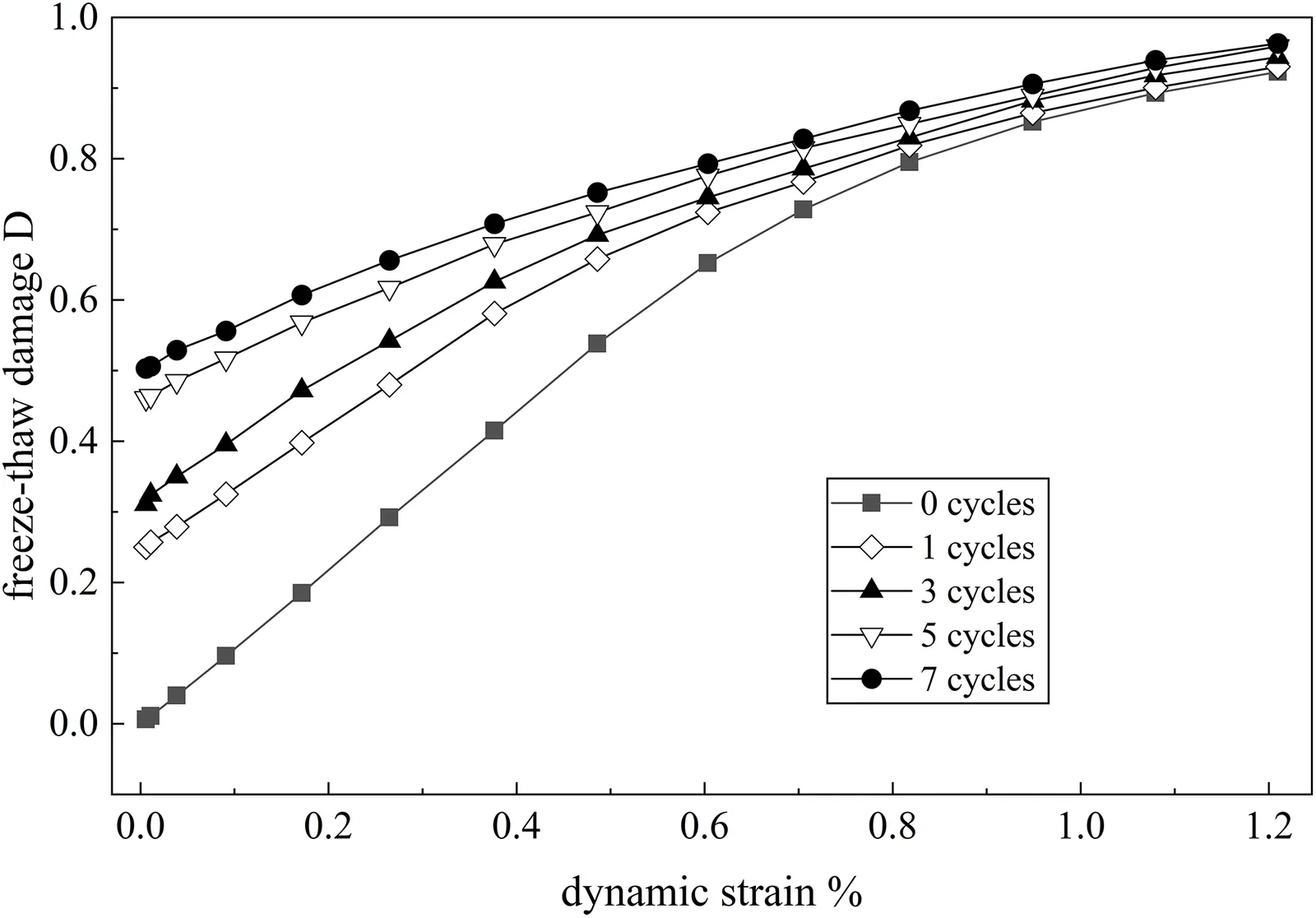
Damage evolution of loess with 6% RHA.

## 6 Mechanisms of soil behavior and microstructural analysis

### 6.1 Scanning electron microscopy analysis

The analysis above indicates that incorporating RHA into loess increases the soil’s dynamic stress and dynamic elastic modulus, reduces the dynamic elastic modulus ratio, and enhances the dynamic damping ratio. This effect primarily results from the reaction of abundant amorphous SiO_2_ in RHA with Ca^2+^ in loess in the presence of water, forming cementitious products, such as calcium silicate hydrate (C-S-H gel) and calcium aluminate hydrate (C-A-H gel). The chemical reaction can be expressed as follows:


$$\begin{array}{c} S_{i}O_{\text{2}}+C_{a}{\left(OH\right)}_{\text{2}}+H_{\text{2}}O\to C-S-H\text{\textasciitilde{}(C-S-Hget).} \end{array}$$ (24)

Incorporating RHA into loess generates cementitious compounds that fill soil pores and bind soil particles, thereby substantially enhancing soil strength. Moreover, RHA surfaces are rich in hydroxyl groups (-OH), which can exchange ions with cations (e.g., Na ⁺ , K⁺) in clay minerals, such as montmorillonite and illite. This ion exchange reduces the thickness of the clay particle double layer, promotes particle aggregation, and improves soil density and structural stability.

SEM analysis of the untreated loess (without RHA) is shown in Fig 13(a). Before vibration, the soil particles exhibited diverse shapes, including plate-like and blocky forms, with varying sizes and a disordered distribution. Large interparticle pores were observed, reflecting the natural microstructure of the loess. The pores varied in size and shape, with some large voids and numerous micropores distributed throughout. These pores constitute the microscopic basis for the loess’s permeability and compressibility, and the large pores serve as channels for the storage and transport of air and water. After vibration, as shown in Fig 13(b), the particle arrangement of the loess was adjusted compared with that of the untreated sample. Some particles shifted, leading to a dense packing. Under triaxial vibration, the particles were redistributed to achieve a stable configuration, resulting in changes in pore size and number. Some of the originally large pores became small or were filled because of particle rearrangement, and the micropores were altered by particle compression, resulting in a reduction in overall porosity. These microstructural changes can considerably affect the physical and mechanical properties, such as permeability and compressibility, of the loess. The particle-to-particle contacts became close, and the number of contact points increased, indicating enhanced interparticle interactions, which may improve the shear strength and other mechanical properties of the loess.

**Fig 13:**
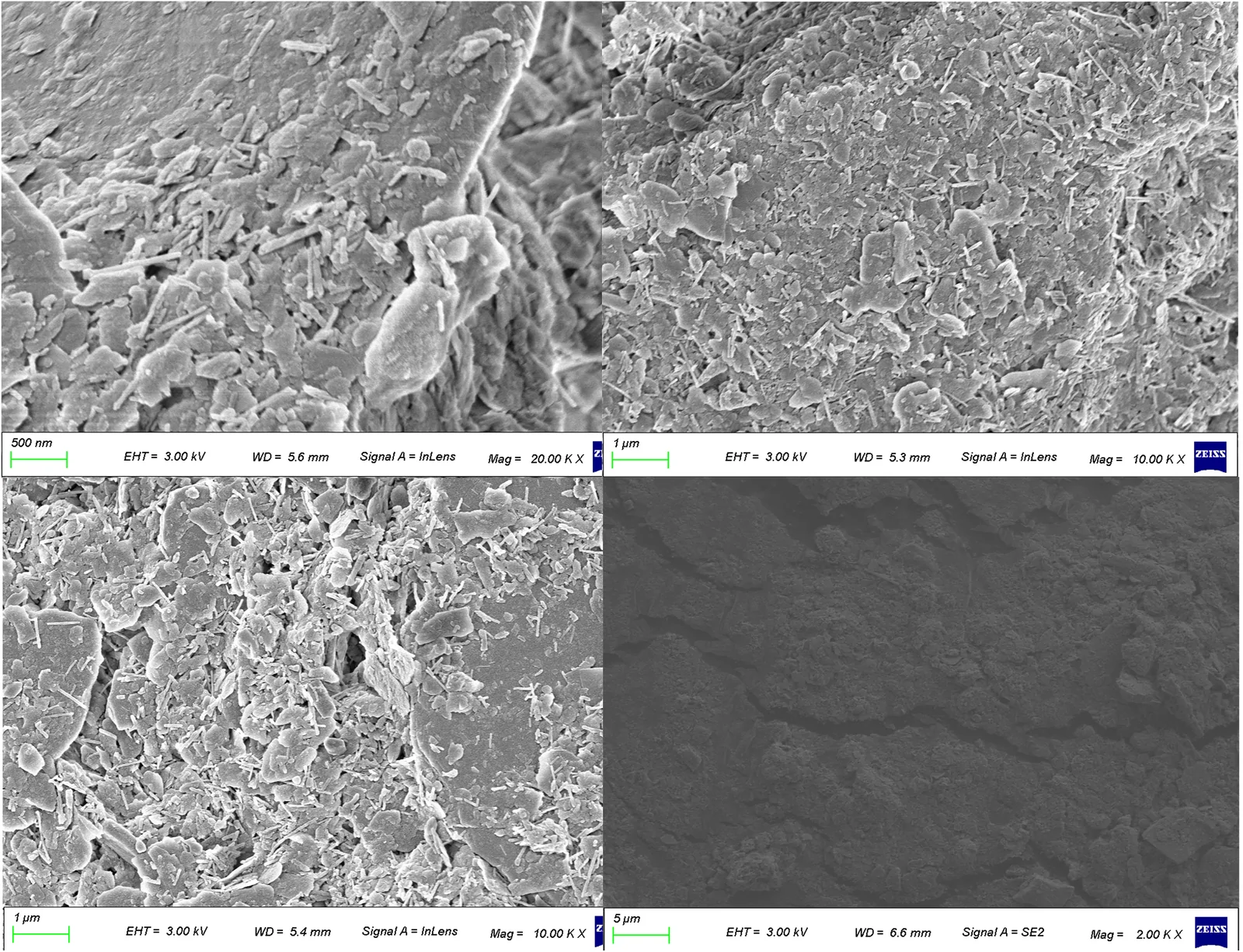
Scanning electron microscopy images. (a) Loess without RHA before vibration, (b) Loess without RHA after vibration, (c) Loess with RHA before freeze–thaw cycles, (d) Loess with RHA after freeze–thaw cycles.

The microstructure of loess mixed with RHA without undergoing freeze–thaw cycles is shown in Fig 13(c). The loess particles were interwoven with the RHA particles. The RHA particles exhibited irregular shapes, mostly elongated or small plate-like forms, and were dispersed among the loess particles. Interparticle pores were present in moderate amounts, varying in size but evenly distributed, reflecting the microstructural state of the RHA-modified loess under unfrozen conditions. Contact and interlocking between RHA and loess particles were observed, indicating physical interactions that likely contributed to the overall cohesion of the soil. This structural arrangement may have partially influenced the mechanical and physical properties of the loess.

The microstructure of RHA-modified loess after freeze–thaw cycles is shown in Fig 13(d). Notable changes were observed compared with the unfrozen state. The number of pores increased, and the pore sizes enlarged, resulting in a loosened soil structure. This result was due to the repeated freezing and thawing during the cycles, where water expansion upon freezing and contraction upon thawing disrupted the soil structure, weakened the interparticle connections, and opened existing pores or formed new ones. Some RHA particles exhibited altered contact with loess particles, showing relative displacement or detachment. The loess particles also experienced partial movement and redistribution, indicating that the freeze–thaw cycles reduced interparticle interactions and compromised the stability of the soil structure.

### 6.2 X-ray diffraction analysis

As shown in Fig 14(a), the mineral composition of natural loess primarily consisted of quartz, calcite, and clay minerals, with the clay minerals mainly comprising illite and montmorillonite. Quartz accounted for 60%, calcite for 27%, and clay minerals for approximately 20%. Other minor minerals, such as brucite, accounted for less than 10%. After the incorporation of RHA into the loess, as shown in Fig 14(b), the quartz content decreased to 40%, and the calcite content declined to approximately 5% primarily because of Ca participation in pozzolanic reactions. New phases, including zeolite and feldspar (~20%), as well as amorphous carbon were formed, and clay minerals were reduced to about 10%.

**Fig 14:**
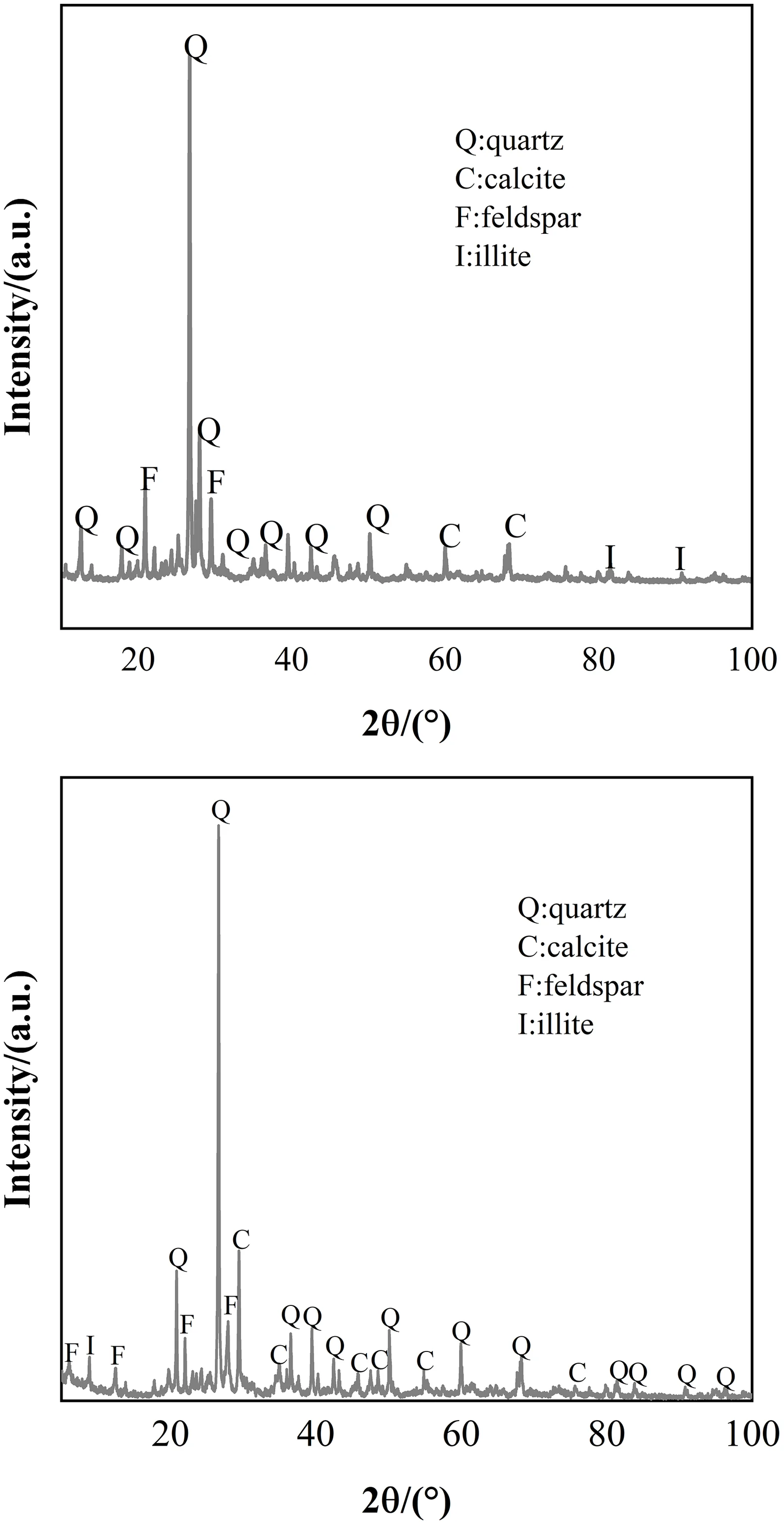
Xray diffraction results. (a) Untreated loess without additives, (b) Loess mixed with rice husk ash.

The primary component of RHA is amorphous SiO_2_. Its addition effectively introduces a large amount of amorphous matrix into the loess, thereby diluting the relative mass fraction of the original crystalline minerals, including quartz, feldspar, calcite, and clay. Residues from RHA combustion contain a small amount of K_2_O, and K ⁺ can undergo interlayer cation exchange with the clay minerals in loess, such as montmorillonite and illite. For montmorillonite, the original interlayer Na⁺ and Ca^2^ ⁺ ions are replaced by K ⁺ , causing the interlayer spacing to shrink from an expanded to a dense state. For illite, K⁺ further stabilizes the interlayer structure and enhances crystallinity, but the overall peak intensity decreases because of the dilution effect of RHA.

Potential chemical reactions include pozzolanic reactions, which are weak and proceed slowly at ambient temperature. The highly reactive amorphous SiO_2_ in RHA can react with Ca^2^⁺ (from calcite dissolution) and Al³⁺ (from clay dissociation) in loess to form low-crystallinity aluminosilicates, consuming part of the original calcite.

The SEM observations and XRD phase analysis revealed that at low RHA contents (0%–6%), amorphous SiO_2_ with high reactivity filled the intergranular voids of loess and participated in pozzolanic reactions to form C–S–H cementitious products, thereby strengthening interparticle bonding. Meanwhile, the porous structure of RHA facilitated the adsorption of free water in the soil, weakening frost heave-induced structural damage. The three synergistic microscale effects collectively enhanced soil compactness and structural integrity. This microstructural evolution directly explains the macroscopic behavior, namely, the gradual increase in dynamic elastic modulus and improved resistance to cumulative deformation after freeze–thaw cycles, with the optimal improvement being observed at an RHA content of 6%. When the RHA content increased to 9%, excessive addition of lightweight and noncohesive RHA disrupted the continuous stress-bearing skeleton of the original loess, generating numerous weak soil–ash interfaces. The available soluble calcium in the soil became insufficient to react with excess reactive silica, leading to the persistence of unreacted inert RHA particles and inherent structural defects. Under the same compaction energy, soil porosity increased, and freeze–thaw-induced cracks and dynamic loading-induced slip progressively developed along these weak interfaces. The coupling of these microstructural defects considerably deteriorated the soil fabric, resulting in a pronounced reduction in macroscopic dynamic performance.

## 7 Conclusions

This study investigated loess from a highway embankment in Xifeng, Gansu Province, by using a GDS dynamic triaxial apparatus. The effects of different RHA contents and freeze–thaw cycles on the dynamic mechanical properties of the loess were examined. The main conclusions are summarized as follows:

1. Incorporating RHA into loess substantially enhanced enhances the soil’s dynamic stress, dynamic elastic modulus, dynamic elastic modulus ratio, and damping ratio. This finding indicates that adding an appropriate amount of RHA is effective in improving the dynamic properties of loess. However, when the RHA content exceeded 6%, these dynamic parameters decreased to varying degrees, suggesting the existence of an optimal RHA content for maximizing soil performance.
2. Under freeze–thaw cycles, the loess incorporated with RHA exhibited higher dynamic stress, dynamic elastic modulus, dynamic elastic modulus ratio, and damping ratio compared with unamended loess. Both RHA-amended and unamended soils were affected to varying degrees by the free–thaw cycles. After seven cycles, the dynamic properties of the soils generally stabilized, indicating that the effects of freeze–thaw damage had reached equilibrium.
3. On the basis of the experimental results, a coupled SMP–microstructural statistical damage model was established and validated; the model showed excellent agreement with the test data. The model was further applied to calculate the damage evolution of 0% and 6% RHA loess under 0, 1, 3, 5, and 7 freeze–thaw cycles. The predicted damage trends were consistent with the observations, demonstrating the model’s suitability for representing the behavior of RHA-modified loess and providing a theoretical basis for further investigation of its underlying mechanisms.

This study has several limitations. The combined freeze–thaw and dynamic traffic loading tests are time-consuming: specimen preparation, curing, freeze–thaw treatment, and dynamic triaxial testing together take several weeks for each test. Because of these time, equipment, and material constraints, replicate specimens were not tested under every condition. To improve measurement consistency, specimen preparation and testing procedures were standardized, and the original dynamic response curves were reviewed to identify and exclude anomalous signals before analysis. Nevertheless, without replicate specimens, the variability of rice husk ash-stabilized loess under freeze–thaw conditions cannot be quantified, nor can the uncertainty of the calibrated constitutive model be fully assessed. Future work will include replicate tests under each condition to quantify experimental variability and strengthen the model’s validation.

## Supporting information

S1 Data Original Data. (ZIP)
